# Does Positivity Mediate the Relation of Extraversion and Neuroticism with Subjective Happiness?

**DOI:** 10.1371/journal.pone.0121991

**Published:** 2015-03-17

**Authors:** Marco Lauriola, Luca Iani

**Affiliations:** 1 Department of Social and Developmental Psychology, University of Rome ‘Sapienza’, Rome, Italy; 2 Department of Human Sciences, European University of Rome, Rome, Italy; Tianjin University of Technology, CHINA

## Abstract

Recent theories suggest an important role of neuroticism, extraversion, attitudes, and global positive orientations as predictors of subjective happiness. We examined whether positivity mediates the hypothesized relations in a community sample of 504 adults between the ages of 20 and 60 years old (females = 50%). A model with significant paths from neuroticism to subjective happiness, from extraversion and neuroticism to positivity, and from positivity to subjective happiness fitted the data (Satorra–Bentler scaled chi-square (38) = 105.91; Comparative Fit Index = .96; Non-Normed Fit Index = .95; Root Mean Square Error of Approximation = .060; 90% confidence interval = .046, .073). The percentage of subjective happiness variance accounted for by personality traits was only about 48%, whereas adding positivity as a mediating factor increased the explained amount of subjective happiness to 78%. The mediation model was invariant by age and gender. The results show that the effect of extraversion on happiness was fully mediated by positivity, whereas the effect of neuroticism was only partially mediated. Implications for happiness studies are also discussed.

## Introduction

The relations between personality and subjective happiness (SH) are complex and determined by multiple factors [1–3]. Extraversion and neuroticism have often been reported as the most prominent personality traits determining the personal set-point around which one’s SH varies [4–5]. The greater positive affect (PA) experienced by extraverted individuals, as well as more frequent involvement in more satisfying social relationships, have often been regarded as the most likely to account for extraversion-SH relations [6–7]. Likewise, the greater negative affect (NA) experienced by neurotic individuals is deemed as the most likely to account for neuroticism-unhappiness relations [6–7]. However, the association of extraversion with happiness is still significant after controlling for number and type of social activities [8]. Furthermore, studies on happy introverted people [9] have challenged the role of extraversion as a crucial personality factor for happiness.

In the present study, we hypothesize that a consistent set of positive beliefs about the future, the self, and the life [10] account for personality-happiness relationships. Separate positive beliefs like self-efficacy, self-esteem, or optimism have been found to mediate between personality characteristics and subjective well-being [11–13]. A recent discovery that separate positive beliefs share a common general positivity factor [10] led us to test whether this factor can effectively mediate between extraversion, neuroticism, and happiness. In the following section, we first review the relevant literature, and then we present the structural equation analysis with which we tested our mediation hypothesis regarding whether extraversion and neuroticism are related to happiness because of a general positivity factor.

### Personality traits and subjective happiness

Subjective happiness is considered as a broad and comprehensive indicator of subjective well-being (SWB) that is defined from the perspective of the person, beyond its multiple components (i.e., life satisfaction, PA, and NA) [14]. Directly asking people about their happiness as an alternative to averaging life satisfaction and affect ratings has attracted a great deal of research in the SWB tradition [15]. Recent studies showed that the correlations of SH with both life satisfaction and PA (*r*s from .45 to .71, and from .32 to .64, respectively) are not high enough to support the complete overlap of these constructs [16–18]. In sum, SH, life satisfaction, and affect states, although related constructs, cannot be equated.

Concerning the antecedents of SH, scholars agree that the extent to which people are happy depends not only on life circumstances or on intentional activities, but also on personality traits [4–5]. During the past decades, there has been an increasing consensus on the claim that five broad trait domains, the so-called Big Five, account for inter-correlations among large collections of trait descriptor terms and individual difference variables [19]. Well-being studies not only established a link between personality and SH, but they also revealed which of the Big Five domains are more predictive. While SH resulted in correlations of around .50 with extraversion and neuroticism, its associations with other Big Five domains were less remarkable: around .15, .30, and .35 on average for openness, agreeableness, and conscientiousness, respectively [13, 18, 20–22]. These findings are consistent with meta-analytic evidence assessing the relationship between each personality trait and happiness as large for extraversion and neuroticism, medium for conscientiousness and agreeableness, and small for openness to experience [6–7, 23]. Extraversion and neuroticism also deserve greater consideration than the other Big Five domains, since they most directly relate to the experience of enduring PA and NA states, respectively [1, 7].

Traits like extraversion and neuroticism are endogenous dispositions that are strongly affected by a heritable component and emerging since early infancy [24]. Accordingly, in the happiness framework, the stability of traits has been considered as a supporting argument for the so-called *set point theory*, which posits that long-term differences in well-being are due to innate temperamental differences between people [4–5, 25–26]. In addition, twin studies showed that a common genetic ground links extraversion and neuroticism to SWB [27].

While the existing literature has highlighted the importance of heritability for personality-happiness relations, Lyubomirsky et al. [5] defined a model of happiness in which the genetic set point is only one of the factors that affect one’s happiness (up to 50%), assigning an important role (up to 40%) to behavioral, cognitive, and volitional activities that offer the best potential to attain a higher level of happiness. In this regard, Tkach and Lyubomirsky [2] showed that a number of happiness-inducing strategies are related to both personality traits and SH, and they proposed a process model in which both traits and intentional activities have an effect on happiness. For instance, social affiliation mediated between extraversion and happiness, while mental control (i.e., the unsuccessful attempt to escape unpleasant thoughts) mediated between neuroticism and unhappiness [2]. Therefore, not only do extraversion, neuroticism, and happiness have a common inborn temperamental tendency [27], but instrumental processes may also link these traits to happiness [2–3].

In regard to extraversion-happiness relations, the greater participation of extraverts in social activities (e.g., attendance at club meetings, number of daily interactions, experience of warmth and affiliation) has become a popular account [28–29]. However, a recent study has challenged this view based on evidence that extraverted people were still happier than introverted individuals after controlling for social activity variables (e.g., time spent with friends) [8]. Moreover, the same study also suggested that an underlying positive incentive motivational system (i.e., reward sensitivity) might give an explanation for both greater sociability and greater positive emotionality. Studies on happy introverted people [9] showed that extraverted individuals not only reported approximately the same level of happiness as the introverted did, but the amount and type of social behaviors was also only marginally different between groups. Thus, Hills and Argyle [30] concluded that emotional stability was as important as extraversion to determine one’s happiness.

Notably, neuroticism (vs. emotional stability) has often been regarded as the strongest predictor of unhappiness [6, 30–31]. Recent studies have shown that neuroticism facets, like depression and vulnerability, explain significant variance in SWB components and SH [32–34]. However, those who are low on neuroticism are not necessarily high on PA. Tkach and Lyubomirsky [2] concluded that the effect of neuroticism on unhappiness was the one least mediated by behavioral strategies, thus remaining large even after intentional activities were controlled for. Therefore, the process linking emotional stability with happiness is more complex and has not been fully understood [30].

### Positive beliefs and subjective happiness

A different research stream in personality psychology also stresses the importance of positive beliefs, such as self-esteem and optimism, as determinants of one’s health and well-being [35]. Self-esteem refers to the evaluative aspects of self-concept [36]. Specifically, it describes the sense of self-pride and worthiness that people derive from the way they manage important aspects of their life. Not surprisingly, self-esteem predicts relevant life outcomes, such as enhanced initiative and pleasant feelings [37]. In regard to the relationship between self-esteem and happiness, a number of studies reported large and positive correlations, in most cases over .50 [20, 38–39]. However, the predictors of happiness are mood, extraversion, neuroticism, global life satisfaction, and social relationships, whereas self-esteem is predicted by optimism and mastery [40].

Satisfaction with life is considered as the cognitive dimension of SWB [41]. Specifically, some authors have framed life satisfaction as a positive belief about one’s life [10]. Just focusing on the past decade, there are at least 25 empirical studies on the relations between SH and life satisfaction, all yielding positive correlations as large as those assessed for self-esteem with happiness [17–18, 42–44].

Two conceptions of optimism have emerged in the literature. Some authors consider optimism as a personality disposition [45], while others define it as an explanatory style [46]. For instance, optimistic people believe that positive events are caused by permanent, global, and internal circumstances, whereas for pessimistic people, positive events are due to temporary, specific, and external causes. During the past decade, at least six studies have shown a positive relation between SH and optimism with a large effect size [21,44, 47].

Another type of optimistic beliefs that has been investigated in relation with SH and personality traits is general self-efficacy [48–49]. In a recent example, general self-efficacy accounted for the correlation between extraversion and happiness, suggesting a pathway from personality to happiness mediated by a positive belief component [13]. Others [11] have shown that general self-efficacy mediates the relation between character strengths and global life satisfaction.

Self-esteem, optimism, and life satisfaction also share a significant amount of variance. For instance, high self-esteem was significantly correlated with life satisfaction [50]. This finding suggests that positive beliefs have a crucial role in shaping the relation between self-esteem and optimism. Thus, Caprara [51] proposed that a common factor, namely positivity or positive orientation, might account for a large amount of the covariance between self-esteem (EST) and optimism (OPT) with life satisfaction (SAT), with all three constructs corresponding to “enduring knowledge structures about oneself and the world that significantly affect one’s feelings and actions, shape the present and predispose to future experiences” [p. 46].

A later study also presented the positivity scale (P scale), whose reliability, validity, and cross-culturally generalizability were tested throughout five independent studies, one of which carried out with Italian, American, Spanish, and Japanese participants [10]. Specifically, a robust unidimensional structure emerged across independent datasets, languages, and cultures, thus supporting the construct validity of the scale. In addition, the P scale total score was positively correlated with energy, emotional stability, and agreeableness (*r*s = .38, .30, and .29 respectively), while the relations of positivity with conscientiousness and openness were smaller (*r*s = .25 and .19, respectively) [10]. The same study also reported a large correlation (*r* = .73) of positivity with life satisfaction. Thus, since the P Scale was correlated with both traits and well-being measures, it has the potential to mediate between these variables.

In sum, the literature has suggested that the interplay of personality traits and positive beliefs contribute to one’s happiness [1], stimulating research efforts to investigate by which process extraversion and neuroticism affect happiness [1, 52]. The relation of positive beliefs with happiness has been hypothesized, but not yet demonstrated in a mediation analysis by using a direct measure of positivity [29]. However, separate positive beliefs (e.g., general self-efficacy, positive automatic cognitions) mediated the relations between personality traits or character strengths and SWB [11, 13, 53].

### Aims of this study

In this study, we bring together a set of constructs relating to positive beliefs and aim to show how these constructs, namely positivity, may jointly contribute to mediate the relation between personality traits and SH. In particular, we hypothesize that the relations of extraversion and neuroticism with SH might be mediated by one’s tendency to evaluate life circumstances as good, namely through positivity. At a more general level, as a main contribution of the present study, we aim to frame general positivity as a social-cognitive variable by which extraversion and neuroticism may affect one’s SWB.

## Methods

### Participants

A total of 504 Italian participants aged 20 to 39 years old (mean age 30.7 years, 50.0% females) and 40 to 60 years old (mean age 49.4 years, 50.0% females) participated in a study presented as a citizen satisfaction survey. Participants were recruited from public places (e.g., streets, railway stations), or from places open to the public (e.g., senior centers) by a trained interviewer. Before obtaining verbal consent, participants received information about the study aims and characteristics. Subjects completed a set of questionnaires and were informed that the participation would have taken about 10–15 minutes. About 10% of the subjects refused to participate. Those who agreed were required to provide their socio-demographic characteristics (e.g., gender, age, education, marital status, and so forth). This was a convenience sample that had an established quota of participants by age and gender that were defined according to the Italian population pyramid. Table 1 reports sample descriptive statistics.

**Table 1:** Sample Descriptive Statistics.

Socio-demographic factors	Levels of socio-demographic factors	*n* (%)	SHS
			*M* (*SD*)
**Gender**	Male	252 (50.0)	4.85(1.13)
	Female	252 (50.0)	4.72 (1.28)
**Age**	20–39	252 (50.0)	4.88 (1.23)
	40–60	252 (50.0)	4.69 (1.19)
**Marital Status**	Married	289 (58.5)	4.89 (1.16)
	Unmarried	162 (31.2)	4.65 (1.27)
	Separated/Divorced	21 (4.1)	4.67 (1.20)
	Widow	6 (1.1)	4.33 (1.27)
	Other/Unspecified	26 (5.1)	4.75 (1.33)
**Education**	Elementary	12 (2.4)	4.83 (.74)
	Middle School	116 (22.3)	4.64 (1.18)
	High School	273 (55.2)	4.88 (1.20)
	University	98 (19.1)	4.70 (1.33)
	Other/Unspecified	5 (1.0)	5.05 (1.05)
**Occupational Status**	Employed	338 (67.8)	4.83 (1.17)
	In search of employment	30 (5.3)	4.28 (1.35)
	Housewife	37 (7.4)	4.83 (1.17)
	Student	33 (6.3)	4.60 (1.38)
	Retired	16 (3.3)	5.02 (1.30)
	Temporary worker	23 (4.4)	4.63 (1.17)
	Other/Unspecified	26 (5.4)	5.01 (1.33)

SHS: Subjective Happiness Scale.

### Measures

#### Personality

Personality traits were measured using the Big Five Inventory (BFI) [54]. Respondents rated 44 short-phrase items starting with the sentence, “I see myself as someone who…” on a 5-point scale (1 = *disagree strongly*, 5 = *agree strongly*). The BFI subscales assessed Extraversion (eight items), Agreeableness (nine items), Conscientiousness (nine items), Neuroticism (eight items), and Openness to experience (ten items). The scores of each scale were computed by taking the mean of the respective items.

#### Positivity

Positivity was measured by the P scale [10]. Respondents rated eight items on a 5-point scale (1 = *strongly disagree*, 5 = *strongly agree*). The sample items are: “I have great faith in the future” (optimism item); “I feel I have many things to be proud of” (self-esteem item); and “I am satisfied with my life” (satisfaction with life item). Higher scores reflect greater positivity.

#### Subjective Happiness

Happiness was measured using the Subjective Happiness Scale (SHS) [14, 55]. Respondents rated four items on different Likert scales, each ranging from 1 to 7. The first item asks respondents how happy they are (1 = *not a very happy person*, 7 = *very happy person*). The second item asks respondents how happy they are in comparison to their peers (1 = *less happy*, 7 = *more happy*). The other two items ask respondents to what extent a description of prototypically happy and unhappy individuals applies to them (1 = *not at all*, 7 = *a great deal*). Higher scores reflect greater happiness.

### Ethics statement

Verbal consent instead of written consent was used to ensure greater confidentiality for participants. Signatures would provide a link between the participant and the study, so individuals who preferred anonymity would have been potentially precluded from participating. Interviewers documented participant consent by signing and dating the informed consent forms after obtaining verbal consent. Ethical approval for the study and for the verbal consent procedure were obtained from the ethical review board for psychological research of the European University of Rome (N. 002).

### Data analyses

Structural equation modeling (SEM) is a family of statistical procedures that permit testing of an a priori model by specifying which variables are assumed to affect other variables and the direction of these effects [56]. In SEM techniques, latent variables are typically used to represent hypothetical constructs that are inferred from a number of interrelated observed variables. The linear relation among latent variables and their respective set of observed variables represent a theoretical model that was hypothesized at the start of the analysis. The analysis aims to test whether the model is supported by the observed data in terms of statistical fit. Mediation analysis is used in SEM to assess whether a variable, e.g. positivity, is intermediate between two other variables (e.g., personality traits and well-being). This technique is frequently used in psychology to discover the mechanism, namely the mediational process, underlying the relation between two variables. The relations between variables are estimated by path coefficients which are partial regression beta weights.

Usually, SEM procedures involve the testing of alternative models in which more than one a priori model is available. In this study, we compared a general mediation model, in which extraversion and neuroticism had both direct and indirect effects on happiness, to a more restricted model, in which non-significant direct or indirect effects of each trait on positivity and happiness were set to zero. The extent to which the more restricted model fits the data, as the general model does, can be used to reveal whether positivity is a full or partial mediator of the hypothesized relations. Last, we tested whether the mediation relations change in particular sub-groups, such as age and gender, whose effects on personality traits and SH have been documented [57–59]. In doing so, we carried out a multi-group analysis using a special form of moderation analysis in which a dataset is split based on the levels of the variable of interest that might potentially affect the strength of associations between independent, mediator, and dependent variables. In this analysis, the same model is simultaneously fit to different sub-group data (e.g., males and females) under the constraint that model’s parameters are invariant across groups.

In the present study, a structural model with two exogenous latent variables (i.e., extraversion and neuroticism) and two endogenous ones (i.e., positivity and SH) was tested by EQS 6.1 [60]. Each latent variable was defined by item parcels, each of which is a composite score reflecting a set of homogeneous items [56]. Parceling is commonly used in SEM to have more parsimonious and reliable sets of observed variables (e.g., the score reliability of parcels is generally greater than that for the individual items), instead of analyzing all items of a specific questionnaire. Moreover, parceling allows for more stable parameter estimates and proper solutions of model fit [61]. For parcels of extraversion (i.e., E1: talkative, generates enthusiasm, quite; E2: assertive, shy, reserved; E3: full of energy, outgoing), neuroticism (i.e., N1: depressed, tense, relaxed; N2: worry, moody, stable; N3: nervous, calm), and SH (i.e., SH1: happy in general, description of happy people; SH2: happy in relation with peers, description of unhappy people), we aggregated items in a pseudo-random way (i.e., items #1, #4 and #7 for E1; # 2, #5 and #8 for E2; #3 and #6 for E3). By contrast, for positivity, we aggregated items based on systematic content similarity, resulting in satisfaction (SAT; i.e., satisfied with life, satisfied with myself), optimism (OPT; i.e., faith in the future, hope, future unclear), and self-esteem (EST; i.e., confident, proud of, faith in other’s help) parcels (Table 2).

**Table 2:** Descriptive statistics for parcels used in structural equation modeling.

Domain	Parcels’ content	*M*	*SD*	Items	First PCA Eigenvalue	Armor’s Theta	Parcel-Domain *r*	Parcel-Domain *r* corrected for overlap
**Extraversion**	E1: talkative, generates enthusiasm, quite (-)	3.27	.88	3	1.58	.55	.88	.67
	E2: assertive, shy (-),reserved (-)	2.98	.77	3	1.36	.40	.82	.56
	E3: full of energy, outgoing	3.82	.90	2	1.34	.51	.78	.59
**Neuroticism**	N1: depressed, tense, relaxed (-)	2.80	.94	3	1.64	.59	.86	.64
	N2: worry, moody, stable (-)	3.09	.90	3	1.68	.61	.85	.63
	N3: nervous, calm (-)	3.30	.95	2	1.25	.40	.80	.63
**Positivity**	SAT: satisfied with life, satisfied with myself	3.73	.85	2	1.56	.72	.79	.62
	OPT: faith in the future, hope, future unclear (-)	2.83	.99	3	1.82	.68	.81	.43
	EST: confident, proud of, faith in other’s help	4.00	.75	3	1.65	.59	.78	.55
**Subjective Happiness**	SH1: happy in general, description of happy people	4.56	1.36	2	1.54	.70	.93	.7
	SH2: happy in relation with peers, description of unhappy people (-)	5.01	1.27	2	1.33	.50	.92	.7

PCA = Principal component analysis; E1 = Extraversion parcel 1; E2 = Extraversion parcel 2; E3 = Extraversion parcel 3; N1 = Neuroticism parcel 1; N2 = Neuroticism parcel 2; N3 = Neuroticism parcel 3; SAT = Satisfaction with life; OPT = Optimism; EST = Self-Esteem; SH1 = Subjective Happiness parcel 1; SH2 = Subjective Happiness parcel 2; (-) reverse scored.

Since the efficiency of the parceling strategy depends on parcels homogeneity [61], we tested this assumption for each parcel using Armor’s θ coefficient (or maximized-alpha). Specifically, θ varies from 0 and 1, and it is computed based on the size of the first eigenvalue in principal component analysis of each item parcel, such that the greater the coefficient, the greater the homogeneity [62–63]. As shown in Table 2, θ coefficients yielded fairly high values for parcels. All the parcel-domain correlations were statistically significant and homogeneous, thus showing that the parcels had a good degree of convergent validity with the total domain score to which they belong (e.g., E1, E2, E3 with Extraversion). Taken together, these preliminary analyses show that the parceling strategy produced almost equally unidimensional and convergent indicators for the corresponding latent variable.

The following fit indices were chosen for data analysis: the maximum likelihood chi-square statistic (MLχ^2^), the Comparative Fit Index (CFI), the Non-Normed Fit Index (NNFI), the Root Mean Square Error of Approximation (RMSEA), and the 90% confidence interval around the RMSEA (90% C.I.). Traditionally, a model’s fit can be evaluated by the MLχ^2^ (or by the alternative Satorra–Bentler scaled χ^2^, SBχ^2^, if a robust method for non-normal data is applied). Nevertheless, since any structural model could be rejected if the sample size is large enough, more “practical” indices of fit have been recommended [64]. The CFI and the NNFI are fit indexes which compare the hypothesized model’s chi-square with one resulting from the independence model (i.e., the model assuming that all relationships among measured variables are 0). CFI or NNFI values greater than or equal to .90 demonstrate an acceptable fit, while values greater than .95 indicate a good fit [65]. In contrast, the RMSEA measures the difference between the reproduced covariance matrix and the population covariance matrix, so that sampling variability is controlled. RMSEA values less than .05 show a small approximation error, while values between .05 and .08 reflect an acceptable error of approximation, and values greater than .10 constitute a poor fit of the model [66]. The 90% C.I. point estimate is also commonly reported to indicate the possibility of a close or exact fit.

## Results

### Preliminary descriptive analysis

Table 1 reports the means and standard deviations for SHS broken down by gender, age, marital status, education, and occupational status. No differences in SHS ratings were found for any variables. The SH level (*M* = 4.79) and other statistics were overall consistent with Italian normative data [55].

Table 3 reports the means and standard deviations for all measures assessed in this study, as well as their inter-correlations. Consistent with the literature, the correlations of SH score with extraversion and neuroticism were larger than those with other personality traits in the Five Factor Model [7]. Consistent with Caprara et al. [10], the correlations between positivity with both extraversion and neuroticism were .36 and −.39, respectively. More importantly, SH and positivity scores resulted in the largest correlation among all those reported in Table 3.

**Table 3:** Correlations between Big Five, Positivity and Subjective Happiness.

	*M*	*SD*	1	2	3	4	5	6	7
**1 Subjective Happiness**	4.79	1.21	-						
**2 Extraversion**	3.30	.70	.42	-					
**3 Neuroticism**	3.03	.78	−.50	−.32	-				
**4 Openness to Experience**	3.50	.70	.24	.37	−.15	-			
**5 Conscientiousness**	3.93	.65	.23	.28	−.30	.31	-		
**6 Agreebleness**	3.76	.61	.21	.26	−.29	.27	.31	-	
**7 Positivity**	3.50	.69	.66	.36	−.39	.29	.32	.24	-

All correlations were significant at *p* < .01.

As mentioned, the literature assigns higher priority to extraversion and neuroticism in personality-happiness studies. Our descriptive analysis showed that other traits (e.g., openness to experience) were also associated with happiness. However, the same analysis also revealed that the Big Five scales were inter-correlated. Thus, traits like openness to experience might be related to happiness due to their association with extraversion. To rule this out, we carried out a standard regression analysis that revealed significant incremental validity of both Extraversion (β = −.44, *p* < .001) and Neuroticism (β = −.63, *p* < .001) over other personality factors (β = .14, *ns*, for Openness to experience; β = .02, *ns*, for Conscientiousness; β = .01, *ns*, for Agreeableness) when predicting SH (*F*_5, 502_ = 49.52, *p* < .001; R^2^ = .33).

These findings not only supported the choice to test a mediation model only for extraversion and neuroticism as exogenous variables in our structural analysis, but also showed that the associations of the other Big Five domains with happiness were of limited importance, as reported in the literature [7]. In addition, our preliminary correlation analysis suggested that positivity could possibly mediate the relationship between personality traits (i.e., extraversion and neuroticism) and happiness (Table 3).

### Structural mediation analysis

**Fig 1:**
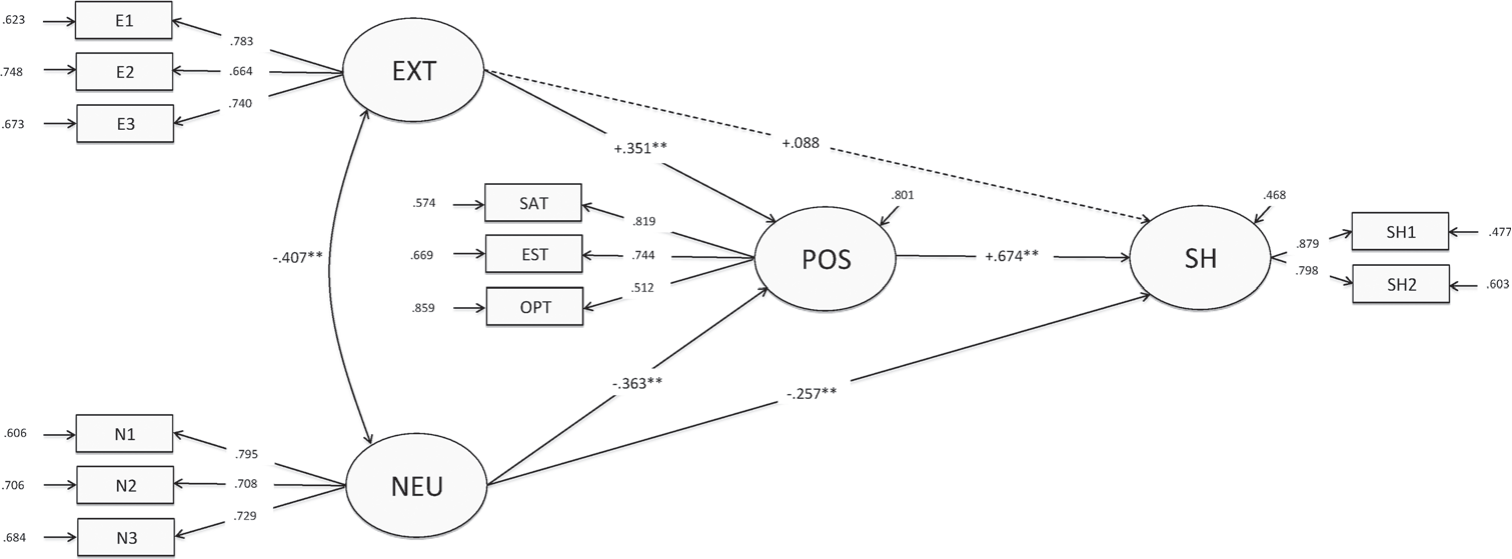
The standardized solution for the hypothesized model. Path coefficients are partial regression beta weights; Dotted line is insignificant relationship; ** *p* < .01; EXT = Extraversion; POS = Positivity; SAT = Satisfaction with life; EST = Self-Esteem; OPT = Optimism; NEU = Neuroticism; SH = Subjective Happiness; E1 = Extraversion parcel 1; E2 = Extraversion parcel 2; E3 = Extraversion parcel 3; N1 = Neuroticism parcel 1; N2 = Neuroticism parcel 2; N3 = Neuroticism parcel 3; SH1 = Subjective Happiness parcel 1; SH2 = Subjective Happiness parcel 2.

The general mediation model in which extraversion and neuroticism had both direct and indirect effects on happiness was statistically significant (SBχ^2^(38) = 105.91; *p* < .001). In spite of this, the inspection of its fit indexes revealed that there was a good fit between the model and the data (NNFI = .95; CFI = .96; RMSEA = .060; 90% C.I. = .046, .073). As shown in Fig. 1, all the parcels had high and significant factor loadings on the latent variables that they were supposed to measure. In particular, factor loadings were greater than .65, .70, .50, and .79 for extraversion, neuroticism, positivity, and SH, respectively. These findings showed that the parceling method was efficient.

Inspection of the structural paths from personality traits to positivity revealed similar path coefficients for both extraversion and neuroticism. The joint effect of personality traits explained 36% of the positivity variance. Furthermore, positivity strongly predicted SH, and the total amount of SH variance accounted for by personality traits and positivity was 78%. Importantly, only the path from neuroticism to SH was significant, while the path from extraversion to SH was not. These latter findings suggest that the effect of extraversion on happiness could be fully mediated by positivity, whereas the effect of neuroticism could be only partially mediated.

To formally test this, we specified a restricted model in which the non-significant direct effect of extraversion on happiness was set to zero. This model not only fitted the data quite well (SBχ^2^(39) = 108.97; *p* < .001; NNFI = .94; CFI = .96; RMSEA = .060; 90% C.I. = .046, .073), but it was also not statistically different from the more general one (ΔSBχ^2^(1) = 3.09; *p* = .08). As we inspected the model’s parameters, we found that the indirect effect of extraversion on SH mediated by positivity was statistically significant (*b* = .27; Wald = 5.22; *p* < .001), as was the case with the paths from extraversion to positivity and from positivity to SH (*b*s = .38 and .72; Wald-s = 6.37 and 11.29, respectively; all *p*s < .001). These findings supported the mediation hypothesis according to the best practices in mediation analysis with latent variables [67–68]. It is worth noting that although different methods adequately control for Type I error, the versatile and computationally easy joint significance test (i.e., testing the statistical significance of each path relating the independent to the dependent variable via mediators) is considered just appropriate enough when only a hypothesis test is of interest, such as in the case of our study [69].

For comparison purposes, we also assessed how much of the SH variance was directly accounted for by personality traits. Thus, we set the paths from extraversion and neuroticism to positivity and from positivity to SH to zero. As expected, the resulting model had a very poor fit to the data, since meaningful paths were omitted (SBχ^2^(41) = 404.73; *p* < .001; NNFI = .73; CFI = .80; RMSEA = .133; 90% C.I. = .121, .144). However, this model showed that neuroticism was the best predictor of SH (*b* = −.50), while extraversion had a lower effect size (*b* = .32). Taken together, neuroticism and extraversion accounted for about 48% of SH variance.

### Multi-group moderation analysis

Age and gender are deemed as personal factors that might affect one’s happiness, as well as extraversion and neuroticism levels [57–59]. As a final step, we tested whether the hypothesized mediation relations were invariant for different age and gender groups. Accordingly, a first multi-group moderation analysis was based on two separate groups, each comprising 252 males or females, respectively. Then, a second multi-group analysis was carried out with two different groups, each comprising younger (N = 252; age range 20–39 years) or middle-aged adults (N = 252; age range 40–60 years).

According to Byrne [70], the initial step in a multi-group analysis requires only that the same structural parameters be specified in different groups (i.e., configural invariance). In our case, such structural parameters were the path coefficients relating extraversion and neuroticism to positivity, positivity to SH, and neuroticism to SH (see Fig. 1). Then, the fit statistics of the estimated model served as a baseline with which more stringent types of invariance can be compared, typically imposing equality constraints to structural regression paths and covariances.

The analysis carried out by gender groups supported the configural invariance hypothesis (SBχ^2^(78) = 165.61; CFI = .93; NNFI = .95; RMSEA = .067; 90% C.I. = .053, .081). As expected, the model with equality constraints imposed to structural coefficients also fitted the data quite well (SBχ^2^(83) = 168.26; CFI = .94; NNFI = .95; RMSEA = .064; 90% C.I. = .050, .078), and, more importantly, it did not differ from the configural invariance model (ΔSBχ^2^(5) = 2.92; *p* = .71). Likewise, the analysis carried out by age groups supported the configural invariance hypothesis (SBχ^2^(78) = 152.21; CFI = .96; NNFI = .94; RMSEA = .062; 90% C.I. = .047, .076). The model with equality constraints imposed to structural coefficients also fitted the data well (SBχ^2^(83) = 161.14; CFI = .96; NNFI = .94; RMSEA = .061; 90% C.I. = .047, .075). Again, no statistically significant scaled chi-square difference between the restricted model and the configural invariance model was found (ΔSBχ^2^(5) = 9.00; *p* = .11). These findings rejected the moderation hypothesis, so we can conclude that the mediation model seems plausible for both men and women, as well as for young and middle-aged participants.

## Discussion

Individual differences in happiness level depend on the interplay of life circumstances, intentional activities, and the type of person [4–5]. In this paper, we focused on personality and hypothesized that the relation with SH of stable personality traits, which are characterized by a strong heritable component [19, 71], was mediated by generalized positive beliefs, such as those recently included in the positivity domain [10]. Our structural model, in which extraversion and neuroticism predicted positivity and happiness both directly and indirectly, fitted the data quite well and provided evidence that the hypothesized mediation was a plausible account of otherwise scattered findings.

Earlier studies found large effect sizes for the relation of happiness with extraversion and neuroticism, with sociability and mental health often called in as explanatory factors [13, 18, 20–22, 28, 30–31]. Our findings are consistent with previous research confirming the large correlations typically assessed for extraversion and neuroticism with happiness. Nevertheless, we disclosed another potential pathway from personality to happiness involving generalized positive beliefs.

In this regard, the positivity construct was recently found to account for the covariance of self-esteem, optimism, and life satisfaction [10], each of which also had an established link with SWB and happiness [17–18, 20–21, 39, 42–44, 47]. In our study, a latent variable representing the positivity construct fully mediated the relation of extraversion with happiness. Accordingly, it seems plausible that extraverted people are more likely to be happy than those with different personality types because they are more apt to approach life with greater confidence and positive attitudes. Boehm and Lyubomirsky [72] already suggested that happy individuals are those who view the world more positively and in a happiness-promoting way. Our findings are indeed fully consistent with this view and demonstrated that people’s appraisals of themselves, their life, and their future [51] are fundamental cognitive mechanisms affecting one’s happiness. In addition, a positive approach to life adds to the happiness increasing strategies described in the literature [2].

In keeping with meta-analytic reviews of personality and happiness [6–7], our structural analysis confirmed that neuroticism (vs. emotional stability) was the best predictor of unhappiness. In this regard, scholars have also emphasized the role of mental health as mediator of the neuroticism-happiness relations [30–31]. Accordingly, the significant indirect effect of neuroticism on SH through positivity is also consistent with the view that there are individuals who are low on neuroticism who approach life positively and are indeed happy [9]. However, the effect of neuroticism on happiness was only partially mediated by positivity. These findings are consistent with other studies that have reported partial mediation effects when considering personality processes alike to positivity, such as mental control and cultivating optimism strategies [2–3].

Last, we examined whether the mediation relations were the same for different subpopulations. This multi-group moderation analysis revealed the total invariance of structural paths for male and female participants, indicating that the mediation model seemed plausible for both men and women in the Italian population. Subsequently, we analyzed the invariance of the mediation model by age groups. Also, this test revealed the total invariance of structural paths for young and middle-aged participants. As a whole, these findings showed that the association of extraversion with happiness via positivity held for different subpopulations.

On a more general level, it is worth noting that personality traits like extraversion and neuroticism are typically considered as fixed set points around which happiness can vary [4–5, 25]. Although we collected cross-sectional data, the amount of SH variance accounted for by traits was about 48% (i.e., a percentage in keeping with the set point theory), whereas adding positivity as a mediating factor increased the explained amount of SH up to 78% (i.e., a percentage much above the presumed influence of a temperamental component). We can indeed conclude that our estimates were consistent with the expected amount of happiness variance accounted for by temperamental influences. Furthermore, we showed that one’s level of happiness cannot be explained by traits only, but a positive outlook is also important to keep the highest possible level of happiness. As Tkach and Lyubomirsky [2] concluded, “people are not genetically destined to experience a predetermined amount of happiness” [p. 221]. Rather, social-cognitive processes such as one’s generalized positive beliefs might expand upon the view of personality traits as predictors of happiness.

Before concluding, we should acknowledge some limitations. First of all, the data upon which we have built our mediation model are cross-sectional. Therefore, they provide only correlational evidence. In addition, as some scholars have pointed out, mediation analyses with cross-sectional data can sometimes provide biased estimates [73–74]. To address this issue, the most pressing task for future research is implementing longitudinal studies or studies involving clinical intervention to fully demonstrate a causal relation from personality to happiness through positivity. In relation to this issue, it is worth noting that not only are our statistical estimates consistent with effect sizes reported in the literature [6–7], but there are also theoretical arguments supporting the more primitive status of personality traits over both positivity and SH. As an example, extraversion and neuroticism have a stronger heritable component [24, 71], emerge earlier in life than life satisfaction, self-esteem, or optimism [75], and are definitely less malleable than social-cognitive personality variables or happiness [76–77]. Thus, an interpretation of our findings from happiness to personality via positivity seems less theoretically grounded rather than the other way around.

The second limitation is that our inferences were based on self-report assessments. Recent studies suggested the use of implicit associations or behavioral indicators for studying the causal mechanisms which link personality to well-being [11, 13, 53]. In our view, a second pressing task for future research is using both self-reports and behavioral indicators in studies focused on the relations between personality and well-being. Moreover, future studies might analyze which of the specific personality facets might have a significant effect on well-being via positivity.

Last, our conclusions are based on data collected by a non-representative sampling method. Despite the large sample size, generalization of our findings to the general population is not warranted. For instance, despite having imposed age and gender quotas according to the national census, other demographics were allowed to vary. As an example, the school-degree distribution resembled that obtained from national statistics [78], with the exception of participants with an elementary school degree, who were underrepresented in our data. However, since education level was quite normally distributed in this study, we think that discrepancy with census data did not bias our structural equation analysis, although generalization of course cannot be taken for granted.

Despite these limitations, our findings might have some practical implications for current happiness research. Different from personality traits, the three constitutive elements of positivity (self-esteem, optimism, and satisfaction with life) are more malleable. For instance, self-esteem and optimism can be successfully enhanced by focused psychological interventions [76–77, 79–80]. In addition, our multi-group moderation analysis suggested that happiness-promoting interventions that target one or more of the knowledge structures mentioned are likely to be equally effective for both men and women, as well as for young and middle-aged people.

In summary, the implications of our study are consistent with the positive-activity model [81] that highlights how effective strategies for increasing happiness should take into account not only the features of positive activities (e.g., dosage, variety), but also the features of the person (e.g., personality, efficacy beliefs) and the person-activity fit.
